# A Fast Calibration Method for Pneumotachograph with a 3L Syringe

**DOI:** 10.3390/bioengineering10091053

**Published:** 2023-09-07

**Authors:** Yueqi Li, Xin Qiu, Hao Zhang, Lirui Xu, Saihu Lu, Lidong Du, Xianxiang Chen, Zhen Fang

**Affiliations:** 1 Institute of Microelectronic, Chinese Academy of Sciences (IMECAS), Beijing 100029, China; liyueqi18@mails.ucas.ac.cn; 2 Aerospace Information Research Institute, Chinese Academy of Sciences (AIRCAS), Beijing 100190, China; zhanghao190@mails.ucas.ac.cn (H.Z.); xulirui19@mails.ucas.ac.cn (L.X.); lusaihu20@mails.ucas.ac.cn (S.L.); lddu@mail.ie.ac.cn (L.D.); chenxx@aircas.ac.cn (X.C.); 3 Personalized Management of Chronic Respiratory Disease, Chinese Academy of Medical Sciences, Beijing 100006, China; 4 University of Chinese Academy of Sciences, Beijing 100190, China

**Keywords:** computational fluid dynamics (CFD), dynamic mesh updating technique, pneumotachograph calibration, 3L syringe

## Abstract

The pneumotachograph (PNT), a commonly used flowmeter in pulmonary function diagnostic equipment, is the required frequency calibration to maintain high accuracy. Aiming to simplify calibration steps, we developed a fast calibration system with a commercially available 3L syringe to provide a real output flow waveform. The acquisition of the real output flow waveform is based on the reliable measurement of in-cylinder pressure and the real-time detection of plunger speed. To improve the calibration accuracy, the tapping position for measuring in-cylinder pressure was optimized by CFD dynamic-mesh updating technique. The plunger speed was obtained by tracking the handle of the plunger with a smart terminal. Then, the real output flow was corrected using a compensation model equation. The calibration system was verified by the pulmonary waveform generator that the accuracy satisfied the requirements for respiratory flow measurement according to ATS standardization. The experimental results suggest that the developed method promises the fast calibration of PNT.

## 1. Introduction

Accurate measurement of respiratory flow is vital for the clinical decision-making of chronic obstructive pulmonary disease (COPD) [1,2,3,4]. The pneumotachograph (PNT) is one of the most common respiratory flow measurement elements used for spirometers. Under laminar flow conditions, according to the Hagen–Poiseuille law, the pressure drop ( Δ P) across the resistance of the pneumotachograph is linear to the flow rate (Q) [5,6,7,8]. However, the linear range, i.e., conductance, can be considered as a constant value and is very narrow [9]. The conductance values reduce slightly with increasing flows. So, when the PNT is used as a linear element, obtaining a calibration curve is simple, but the measurement accuracy will be lost. To improve measurement accuracy, PNT needs to use higher-order polynomial calibration curves. The frequency calibration of non-linear PNT, which is required to maintain accuracy [10,11], is generally a complex and time-consuming task, as it usually requires a source of constant airflow. However, reliably accurate reference waveform generators are not widely available.

The 3L calibration syringe has the advantages of being a small piece of equipment, having a low cost, and a simple operation. It is an ideal quality control device commonly used in pulmonary function laboratories. Finding out whether the calibration curve of a PNT device can be obtained with only a 3L syringe has sparked interest among researchers. Since it is difficult to generate a steady flow by manually pushing the syringe, indirect methods have been proposed to obtain the calibration curves of PNT. As early as 1982, Yel et al. [12] developed a simple method named the weighted averaging (WA) technique to obtain the conductance values of PNT. In subsequent research, the calibration curves were supposed as second and third-order polynomials, as proposed by Tang et al. [13], the power law equation, as described by Biselli et al. [14], and polynomials with regularizing penalization added, as developed by Quelhas et al. [15]. On top of these studies, multiple strokes at different flow rates were needed to cover the whole calibration range. A common strategy of the indirect methods of nonlinear calibration of PNT is to find optimal parameters to minimize the error between the known syringe volume (3L) and the volume obtained by integrating the calibrated PNT waveform in each stroke. However, this strategy cannot guarantee the accuracy of the calibration requirements for both volume and flow. As the flow rate through the PNT increases, the flow resistance increases. The gas in the syringe barrel will be compressed (injection) or expanded (withdrawal), which leads to the calibrated PNT measuring volume being less than or more than 3L [16]. Hankinson et al. [17] solved this distortion problem by calculating the compression volume in the cylinder using the adiabatic process of an ideal gas, which requires simultaneous monitoring of the plunger displacement and the absolute pressure of the gas inside the cylinder. On this basis, Cross et al. [18] proposed a syringe potentiometer (SP) for PNT calibration by reinventing a 3L syringe. Volume and flow waveforms obtained from the SP device were deemed accurate surrogates for the actual syringe volume and flows. However, assembling a SP device is not easy. A set of 3D-printed components needs to be additionally manufactured. A draw-wire potentiometer is required to purchase. Finally, a small-bore pressure tap is demanded to drill into the faceplate of the calibration syringes. These additional accessories and complex processes would not only increase costs but also weaken its popularization. To date, none of the above-mentioned methods can be widely used.

In this study, an alternative simplified PNT calibration method was proposed, which can achieve fast calibration without adding accessories and modifying the 3L syringe. This paper is organized as follows. Firstly, in the Method section, there will be a description of the test setup for the experiments and detailed information on the simulation experiments. Secondly, the simulation results of piston movement such as velocity and pressure profiles, the static pressure comparison between origin pressure tapping and new pressure tapping, and the validation of the calibration system are described in the Result and Discussion section. Finally, the research significance of this work based on the achieved results is reported in the Conclusions.

## 2. Methods

**Figure 1:**
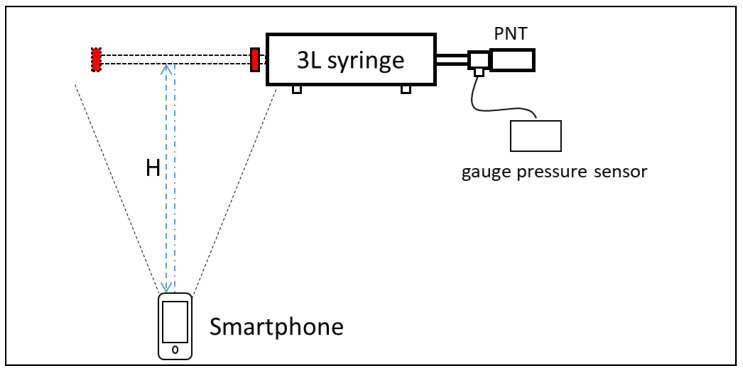
The schematic of the calibration system.

Figure 1 shows the schematic of the calibration system. A 3-L precision calibration syringe (Chest M.I., Tokyo, Japan) was used for this experiment. The typical structure of a commercial 3L calibration syringe is a lightweight piston mounted horizontally in a right cylinder. The total volume inside the cylinder is calculated by the product of the plunger displacement (L) and the area of the syringe piston head (S):

(1) $$V_{syr}=S\times L$$

where the trajectory obtained by the object tracking algorithm is taken as a surrogate for L, given the assumption that in each calibration stroke the movement of the extrusion profile occurs only along the principle axis of the syringe. Since the total volume and area of the syringe piston head are constant, we only need to monitor the length changes of the plunger to obtain the instantaneous volume in the cylinder. The length of the plunger of this 3L calibration syringe is 37.5 cm, and that of other common brands is similar. In practical use, we put these data into the program as the input parameter to ensure the algorithm can apply all commercial calibration syringes. For universality and ease of operation, high saturation colored electrical insulating tape (3M, Saint Paul, MN, USA) was attached to the end of the plunger of a 3L syringe as a mark point for target tracking. The real-time tracking trajectory of the color marker was used as a real-time substitute for the plunger movements. All experiments mentioned in this paper were implemented with a red color code. The trajectory of the plunger translated into real-time changes in the volume of the cylinder in subsequent calculations. The shooting direction of the camera is perpendicular to the plunger of the syringe, as shown in Figure 1. The marked **H** in Figure 1 represents the distance between the rear camera and the plunger. The distance can be arbitrary as long as it satisfies the conditions that the camera shooting range can cover the moving range of the color code. The mobile APP calls the rear HD camera of the mobile phone to shoot video in 1080 P mode with 60 frames (the highest possible resolution and frame rate were chosen to capture the details of the movement of the plunger). During the experiment, the smartphone was placed on a stable base for shooting to avoid camera shaking. According to the experimental requirements, no color was the same or close to the color code in the video background. The gauge pressure sensor and its measurement system were the same as in the previous experiment.

The application in the mobile terminal, which was developed under the integration environment of Android Studio, implemented three functions: (1) to track and save the color code movement trajectory and convert it into a real-time cylinder volume; (2) to obtain, process, display, and save the data of gauge pressure by the Bluetooth communication; and (3) to fit the calibration curve with the real-time gauge pressure data and real output flow data and display it.

In this system, the two factors that had a greater impact on the calibration results are the selection of the tapping position and the deviation caused by gas compression, or the expansion in the cylinder. The selection of the tapping position was analyzed and optimized using the dynamic-mesh updating technique. The deviation caused by gas compression or expansion in the cylinder was corrected using a model equation.

### 2.1. Tapping Position Optimization

A two-dimensional dynamic transient simulation was adopted to simulate the injection and extraction process when the 3L syringe is connected to a PNT, which facilitated observing the whole fluid domain to find the appropriate substitution for the measurement tapping position.

#### 2.1.1. Presumptions

To facilitate the establishment of a mathematical model describing the motion of the cylinder, some presumptions were made for quantitative analysis of the motion process of the plunger:

1. The air in the 3L syringe is set as the ideal gas, which satisfies the ideal gas state equation;
2. The friction between the plunger and the cylinder wall is relatively small and can be ignored;
3. The gas in the cylinder cavity has no heat exchange with the outside world. It is an adiabatic process. The gas temperature is the ambient temperature;
4. Cylinder leakage can be ignored.

#### 2.1.2. Governing Equations

When the boundary is moving, the integral form of the conservation equation on an arbitrary control volume, V, can be written as [19,20,21,22]:

(2) $$\frac{d}{dt}\int_{V}\rho \phi dV+\int_{\partial V}\rho \phi \left(\vec{u}-{\vec{u}}_{g}\right)\cdot d\vec{A}=\int_{\partial V}\Gamma \Delta \phi \cdot d\vec{A}+\int_{V}S_{\phi }dV$$

where ρ is the fluid density, $\vec{u}$ is the flow velocity vector, ${\vec{u}}_{g}$ is the grid velocity of the moving mesh, Γ is the diffusion coefficient, and $S_{\phi }$ is the source term if ϕ . Here, what is used to represent the boundary of the control volume is V. The time derivation term in Equation (3) can be written using a first-order backward difference formula as:

(3) $$\frac{d}{dt}\int_{V}\rho \phi dV=\frac{{\left(\rho \phi V\right)}^{n+1}-{\left(\rho \phi V\right)}^{n}}{\Delta t}$$

where *n* and *n* + 1 denote the respective quantity at the current and next time levels. The (*n* + 1)th time level volume is calculated using:

(4) $$V^{n+1}=V^{n}+\frac{dV}{dt}\Delta t$$

where $\frac{dV}{dt}$ is the volume time derivative of the control volume. To satisfy the grid conservation law, the volume time derivative of the control volume is computed from:

(5) $$\frac{dV}{dt}=\int_{\partial V}{\vec{u}}_{g}\cdot d\vec{A}=\sum_{j}^{n_{f}}{\vec{u}}_{g,j}\cdot {\vec{A}}_{j}$$

where $n_{f}$ is the number of faces on the control volume and ${\vec{A}}_{j}$ is the j-face area vector. The dot product ${\vec{u}}_{g,j}\cdot {\vec{A}}_{j}$ on each control volume face is calculated from

(6) $${\vec{u}}_{g,j}\cdot {\vec{A}}_{j}=\frac{\delta V_{j}}{\Delta t}$$

$\delta V_{j}$ is the volume swept out by the control volume face over the time step Δt .

#### 2.1.3. Dynamic Mesh Method and Boundary Conditions

**Figure 2:**
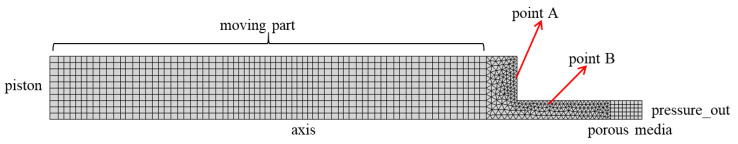
The mesh model of the 3L syringe.

In this case, to characterize the performance of the fluid passing through the PNT, the dynamic mesh was used to simulate the plunger movement and the porous media was used to simulate the PNT. Since the movement of the plunger was a simple directed rigid body motion, the dynamic layering method can be used to update the grid to simulate this form of motion. Therefore, the 3L calibration tube grid was divided into three regions. In order to retain a certain observation area for point A, the moving part did not cover the entire piston stroke. The quadrilateral structured grids were built for moving parts and porous media. The unstructured triangle elements were built for stationary regions. Given the symmetry of the computation domain, to reduce the computation time, half of the 2D calibration syringe geometry with the same physical size was used, consisting of a rigid body of piston on the left and a pressure outlet on the right. The mesh model is shown in Figure 2.

The ANSYS Fluent^®^ (ANSYS 2020 R1) software was flexible for CFD simulation. The users can compile User-Defined Functions (UDFs) according to their specific modeling needs [23]. In the present study, the macros of DEFINE_CG_MOTION were used to specify plunger (rigid body) velocity and displacement. Stationary zones were maintained intact for an update. The following velocity expression was used to simulate the reciprocating motion of the piston:

(7) v=A·2·π·f·sin(2·π·f·t)

where A is the amplitude, f is the frequency, and the maximum velocity is ( 2·π·f·t ). The appropriate A and f values were set to cover the range of detection.

The porous media model in Fluent was used to simulate PNT. By setting the key parameters, the flow characteristics of the fluid through PNT would be consistent with the real data to achieve the purpose of simulation. The key parameters include inertial resistance coefficient, viscous resistance coefficient, and porosity. Based on the calculation method offered by the ANSYS fluent 12.0 Documentation [24], the calculated key parameters are 1.5 × $10^{9}$ , 15, 112, and 0.95, respectively.

**Figure 3:**
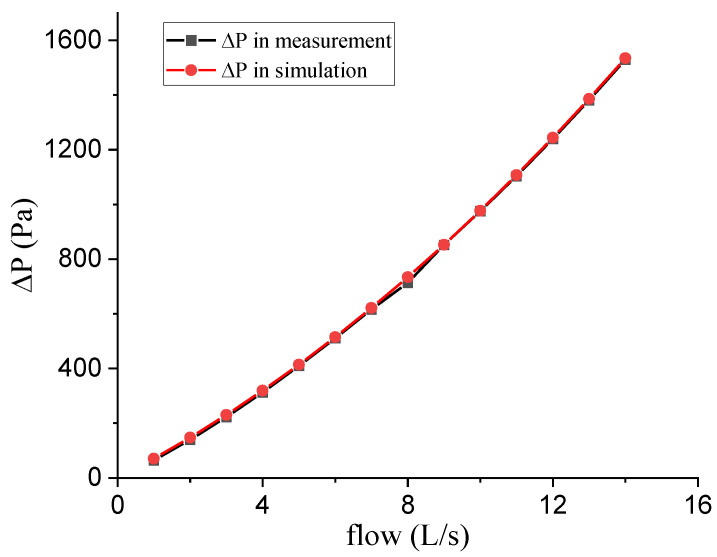
The comparison of numerical simulation and experimental data.

The feasibility verification of the porous media model is shown in Figure 3. The simulation (red line) and measurement (black line) results of 14 groups of flow (from 1 to 14 L/s with a step of 1 L/s) were recorded. It can be seen that the numerical simulation results were in good agreement with the experimental results, indicating that it is feasible to use the porous media model in Fluent to simulate the characteristics of PNT.

### 2.2. Experimental Method

#### 2.2.1. Experiment 1: Validation of Tapping Position

A Fleisch-type pneumotachograph (0–800 L/min range, Piston Medical Ltd. Budapest, Hungary) was used for the experiments. An auxiliary gasket ring with a pressure tap was fabricated using 3D printing technology to connect the PNT to the calibration syringe. We connected the pressure tap to gauge pressure sensors (ABPMRRN010KG2A3, Honeywell, Columbus, OH, USA), attached to a 12-bit ADC (Analog-to-Digital Converting) (NI USB-6008, National Instruments, Austin, TX, USA). On the faceplate of the commercially available 3L calibration syringe (Cubic Sensor and Instrument, Wuhan, China), a small hole was drilled as the pressure tap, whereby the pressure inside the syringe barrel can be real-time monitored by connecting one port of the same type of gauge pressure sensors mentioned above. A primary microcontroller module (CC2640R2F module-based ARM Cortex^®^-M3 core, JieFan Scientific Company, Guangdong, China), which serves as the Bluetooth transmitter at the same time, was used for respiratory signal acquisition and data wireless transmission. The data processing, data storage, and result displaying were completed in an application for smartphones or other mobile terminals. The waveforms of the combined data set were low-pass filtered at 25 Hz with a fifth-order Butterworth design. The sampling frequency was set to 250 Hz.

#### 2.2.2. Experiment 2: Validation of the Calibration System

The object tracking algorithm worked in the application and was implemented using Meanshift [25,26,27,28,29], a ready-to-use function provided by the OpenCV library, and is the iteration tracking algorithm based on a color histogram. The advantages of the target tracking algorithm based on color feature included (1) a small computational cost and (2) the histogram generated by the HSV color space is not more sensitive to light changes than the one generated by the RBG color space. The limitation is that similar colors cannot appear in the background. Compared to a draw-wire potentiometer, the color code tracking method is very simple. This method is not affected by the material and appearance of the 3L calibration cylinder. Although the experiments were carried out indoors, there is no guarantee that the lighting conditions would not change at different times and in different places. Therefore, at the beginning of the experiment, the region of interest (ROI) was retrieved.

**Figure 4:**
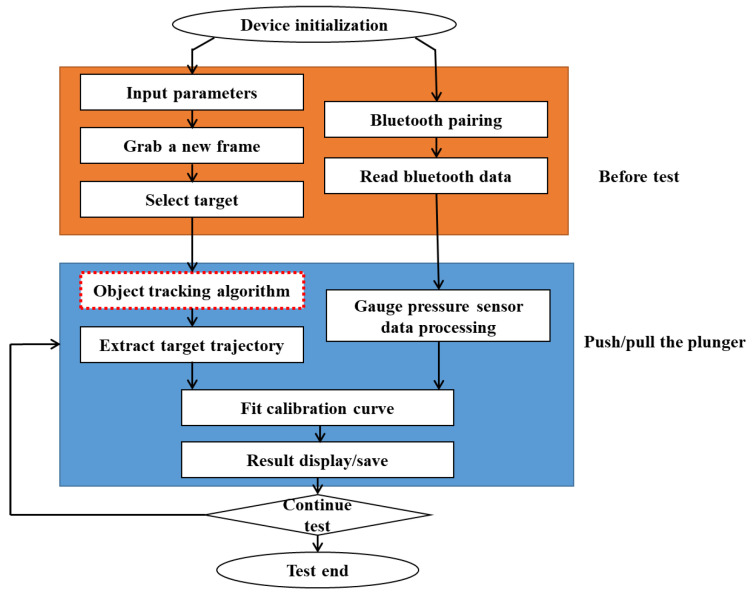
The flowchart of the application.

The flowchart of the app is shown in Figure 4.

Before the tests, the device needed to be initialized. The device Bluetooth was pre-set to the communication mode. Once the Bluetooth pairing was complete, the gauge pressure data began to transmit immediately. Then, the plunger length, frame rate, and resolution settings of the camera were entered as the input parameters. When the camera was ready, the current video frame was captured and the target was selected interactively. Then, the experiment begins with a push-pull plunger at different speeds several times to cover the largest possible flow range. The gauge pressure data processing included extracting gauge pressure sensor data based on the transmitted packet pattern. Finally, calibration curves were fitted using gauge pressure data and real output flow data. The result of the fitted curve was displayed on the screen.

#### 2.2.3. Experiment 3: The Correction for Real Output Flow

**Figure 5:**
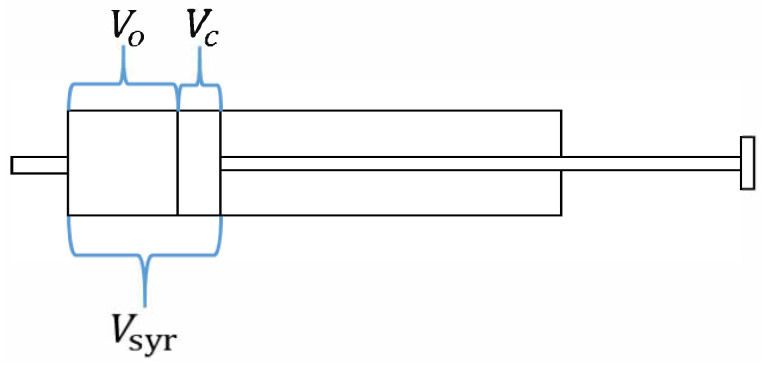
Schematic diagram of gas compression during fast push plunger.

The flow resistance of the PNT increases with the increase in the flow rate. When the flow resistance of PNT is too large to be ignored, gas compression will occur in the cylinder. The compressed volume is $V_{c}$ . In theory, the volume discharged from the 3L calibration syringe was supposed to be $V_{syr}$ , but the actual discharged volume is $V_{o}$ , as Figure 5 shows.

(8) $$V_{syr}=V_{o}+V_{c}$$

According to the adiabatic process of an ideal gas [30],

(9) $$constant=V^{r}P$$

Suppose that the gas compression process is a quasi-static transition from pre-compression with pressure $P_{b}$ and volume $V_{syr}$ to post-compression with pressure $P_{syr}$ and volume $V_{o}$

(10) $$V_{syr}{}^{r}P_{b}={\left(V_{c}+V_{o}\right)}^{r}P_{b}=V_{o}{}^{r}P_{syr}$$

where $V_{syr}$ is the instantaneous volume within the cylinder, including connecting tubing, $P_{syr}$ is instantaneous pressure in the cylinder measured at the front tap, $P_{b}$ is barometric pressure, and γ is the ratio of the molar heat capacity at a constant pressure to the molar heat capacity at a constant volume (for air, γ is 1.4).

Finally, we obtain the gas compression ( $V_{c}$ ) formula for the instantaneous volume loss:

(11) $$V_{c}=V_{syr}\times \left[{\left(\frac{P_{syr}+P_{b}}{P_{b}}\right)}^{\frac{1}{\gamma }}-1\right]$$

The pump-corrected displacement volume was $V_{syr}+V_{c}$ during the injection process, while it was $V_{syr}-V_{c}$ during the withdrawal process. The first derivative of the pump-corrected displacement volume was the syringe flow, i.e., real output flow.

#### 2.2.4. Experiment 4: Validation of Calibration Accuracy

The pulmonary waveforms generator (PWG-33, Piston Medical Ltd. Budapest, Hungary) with high accuracy and fidelity was used as standard equipment to depict the real calibration curves of the PNT. The PWG-33 can provide the waveforms with 0.5% and 0.2% accuracy of flow and volume, respectively. The 14 sets of flow waveforms (from 1 to 14 L/s at a step of 1 L/s) were used to calibrate. The pressure drop was recorded by the measuring equipment with differential pressure transmitters (ABPMRRN010KD2A3, Honeywell, Columbus, OH, USA). The polynomial curve fit was applied and a linear regression equation was obtained with a correlation coefficient ( $R^{2}$ ) of 0.9995 as the standard reference flow curve.

## 3. Results and Discussion

### 3.1. Tapping Position Optimization Results

**Figure 6:**
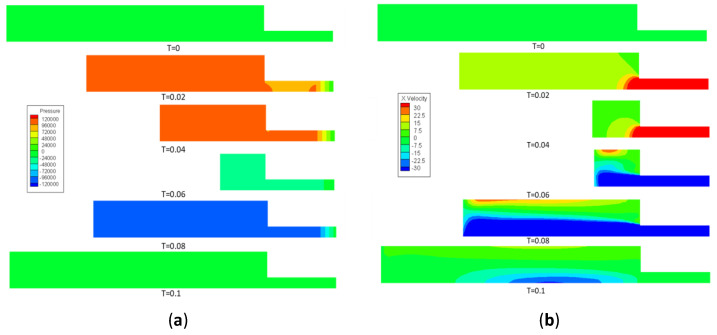
Static pressure distribution (Pa) (**a**) and velocity distribution (m/s) (**b**) change with the movement of the piston.

Point A was the pressure tapping position that was commonly selected in previous studies. Point B was the upstream point of the PNT. Both points are marked out in Figure 2. During the movement of the piston, the pressure, and the velocity changes in the fluid domain are shown in Figure 6. It can be seen from the pressure distribution that a uniform pressure gradient decreases at the porous media in the injection stroke. The static pressure at point A in the fluid domain was higher than that of point B. In the withdrawal stroke, except for the porous media, the static pressure in the fluid domain was equal everywhere. It can be seen from the velocity distribution that the velocity of the center changes significantly faster than that of other positions, which conforms to Bernoulli’s equation. When the piston was pushed, the gas in the cylinder was pushed to the open port of the cylinder. The backflow was formed at A, which leads to the instability of the pressure signal. In the withdrawal stroke, a similar phenomena occurred. Therefore, in simulation results, point B is a more suitable location for the tapping position.

### 3.2. Experiment 1: Validation of Tapping Position

**Figure 7:**
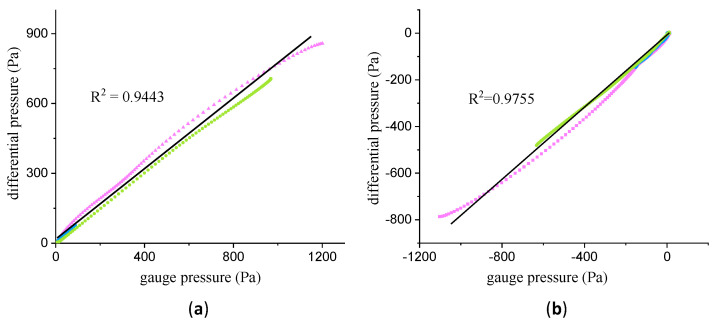
The correlation between the differential pressure and gauge pressure within the push strokes (**a**) and the pull strokes (**b**).

To test the correlation of the gauge pressure between points A and B, we conducted three tests with a low, medium, and high-speed push-and-pull plunger, respectively. Figure 7a,b show the regression of the line of the gauge pressure between points A and B within the push strokes and the pull strokes. The three three-color curves represent three different speed experiments. The correlation coefficient between these two is $R^{2}$ = 0.9443 for the push stroke and $R^{2}$ = 0.9755 for the pull stroke, respectively. It can be seen that there were differences between the measured data and the simulation data in the process of withdrawal. The static pressure of points A and B was equal everywhere in the simulation data, but not in the measured data. The reason is that the simulation experiment was implemented under ideal conditions. In fact, during the pull process, violent backflow occurs at point A, resulting in different static pressures at the two points A and B in the actual measurement. As demonstrated in the previous experiment, we found that the stability of the gauge pressure data collected at point B was better than that of point A. The correlation exists in the gauge pressure data of two points. So, we tried to take advantage of the correlation to obtain the in-cylinder pressure using the gauge pressure measured at point B.

### 3.3. Experiment 2: Validation of the Calibration System

In this paper, we utilize the MeanShift tracking algorithm to detect and track signs using a camera phone. We aimed to implement a robust, lightweight tracking algorithm on devices with low processing capabilities. We captured the red color code as a substitute indicator for plunger movement. Thus, we used the target tracking algorithm to obtain the velocity and displacement of the plunger, after calculation, which is converted into a real-time change of volume in the cylinder. In order to test the accuracy of the color-based object tracking algorithm, 10 complete injection and withdrawal strokes were collected. Whether push stroke or pull stroke, the calculation result should be 3L. The results are shown in Table 1. The average and standard deviation of these 10 tests was 3.0106 ± 0.003715 and 2.9994 ± 0.003093 for push stroke and pull stroke, respectively. The maximal error found in the trials was 0.47%.

### 3.4. Experiment 3: The Correction for Real Output Flow

In the intelligent terminal, the target tracking trajectory data and differential pressure sensor data were recorded simultaneously. The real-time compressed gas (or expansion gas) volume in the cylinder within the injection stroke (or withdrawal stroke) was calculated by substituting the two into Formula (11). Then, the actual discharged volume was calculated by substituting the compressed gas volume into Formula (8). Thus, the calculated actual discharged flow was the recognized real output flow in our system. The calibration curve given by our system was obtained by the real-time real output flow and the real-time difference pressure data fitting.

**Figure 8:**
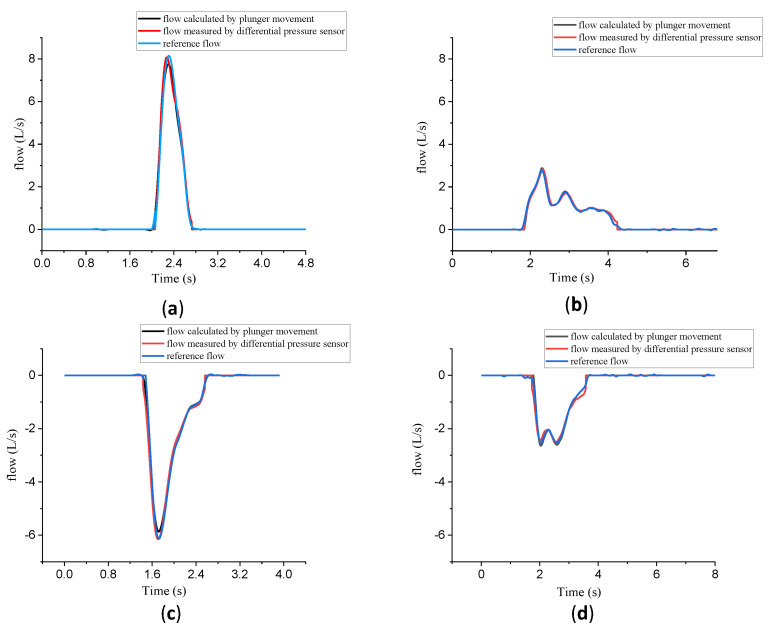
The comparison of push stroke high speed (**a**) and low speed (**b**) and pull stroke at high speed (**c**) and low speed (**d**).

To verify the reliability of the above calculations, two sets of experiments with a 3L syringe emptied through the flow sensors at different speeds were conducted. Figure 8 shows the comparison of the real-time flow measured by different data sources in a push stroke at fast (a) and slow (b) speeds and in a pull stroke at fast (c) and slow (d) speeds. There are three colored lines in Figure 8, below. The red line is the real-time flow converted from the calibration curve of PNT provided by PWG. The blue line is the real output flow calculated by the formulas. The black line is the product of the velocity of the real-time plunger movement and the cross-sectional area to facilitate comparison with the other two curves.

It can be seen from Figure 8 that the real output flow and the actual discharged flow were the same in the low-speed flow experiments, but different in the high-speed flow experiments. After the compression (or expansion) volume correction, the real output flow was highly consistent with the actual discharged flow throughout the experimental flow range, which agreed with the above-mentioned theory.

**Figure 9:**
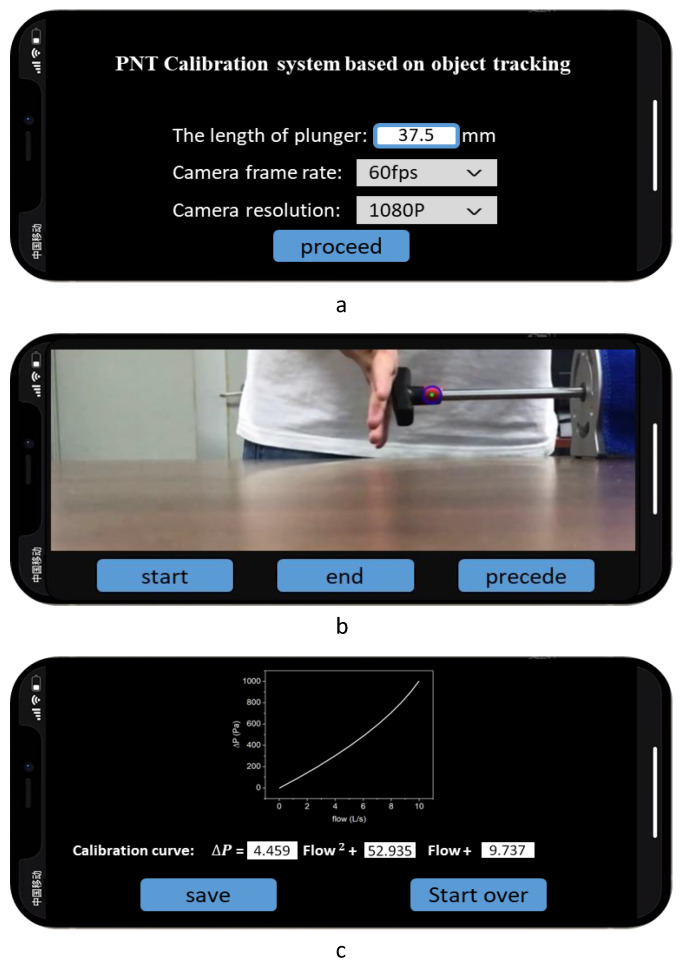
The displays of the smartphone app following one calibration test using the developed calibration system. (**a**) The initial screen including parameter input; (**b**) the real-time display of target tracking; and (**c**) the calibration curve and the calibration formula of the test.

Figure 9 shows several smartphone app interfaces that the terminal APP would display in a complete test, which includes the parameter input (Figure 9a), the real-time display of target tracking (Figure 9b), the calibration curve, and the formula of the test (Figure 9c). The blue circle in Figure 9b is the tracking window, which identifies the area of maximum pixel distribution. The trajectory of the center of the circle, the green dot, is regarded as that of the plunger. It is proven that the system meets the real-time processing requirements to ensure high-precision calibration and stable operation.

### 3.5. Experiment 4: Validation of Calibration Accuracy

**Figure 10:**
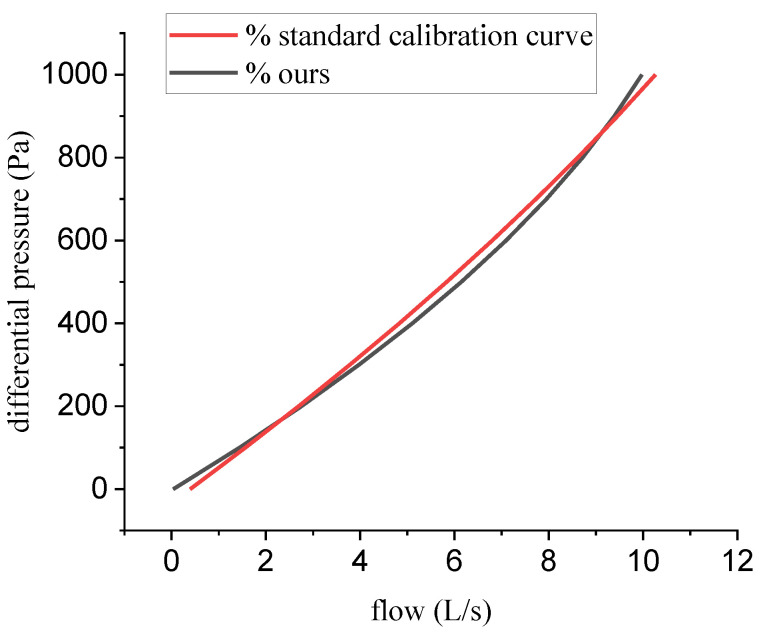
Comparison of standard calibration curves and ours.

Figure 10 shows the comparison between the calibration curve measured by PWG-33 and the one obtained by our system. As we can observe in Figure 10, our curve was generally consistent with the standard ones. Our calibration curve was slightly lower than the standard one at low and high flow rates, and slightly above the standard curve at medium flow rates. To test the accuracy and reliability of flow measurement when PNT adopted our calibration curve, we used a pulmonary waveform generator PWG-33 to create a set of 20 waveforms with different speeds and directions for the PNT performance test. The peak flow data were recorded to measure whether the flow error obtained by our calibration curve meets the requirements within the flow measurement range.

**Figure 11:**
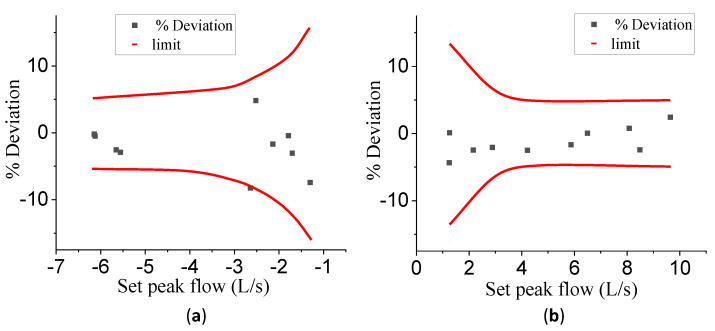
The distribution of accuracy of the test results in the pull stroke (**a**) and push stroke (**b**).

According to the standardization of spirometry [10], the flow rate must be measured with an accuracy of at least ±5% of reading or ±0.200 L/s; whichever is larger. The red lines in Figure 11 represent the upper and lower limits of the accuracy requirement. As Figure 11 depicts, the deviation of the test results was distributed between the upper and lower limits. All these test results indicate that the accuracy of PNT using our calibration curve meets the spirometer standards.

**Figure 12:**
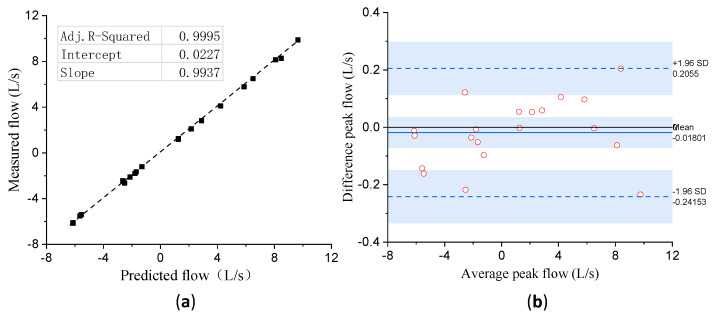
Correlation (**a**) and Bland–Altman plots (**b**) of flow measured from standard waveforms discharged by PWG-33.

The statistical test results are shown in Figure 12. Figure 12a shows the regression line between the peak flow values calculated by our calibration curve and the set values, and the correlation coefficient $R^{2}$ = 0.9995. Here, the Bland–Altman plot was used to describe an agreement between the two. The comparison results were described as a scatter diagram XY, in which the Y axis was the difference between the measured peak flow values and the set values, and the X axis refers to the average of the two. The bias (mean) and limits of agreement (bias ± 1.96 × SD) are represented by a blue solid line and blue dash-dot lines, respectively. It can be seen from Figure 12b, that most points were within the bounds of agreement, which indicates the peak flow values calculated by our calibration curve can be evaluated as a very good agreement with the set values.

## 4. Conclusions

The calibration system based on target tracking reported in this paper provides an easier-to-promote solution for the laboratory calibration of PNT-type flow sensors using a 3L calibration syringe. According to the experimental results, the calibration curve provided by the system is in good agreement with the one obtained by the traditional method. The measurement accuracy of flow using the calibration curve provided by our system is comparable to that of commercially available flowmeters. Given that our proposed system has the advantages of simple structures and strong portability of the intelligent terminal, the calibration system will provide an attractive choice for laboratory PNT flow sensor calibration to improve the quality of flow data for both research and clinical applications.

## Figures and Tables

**Table 1:** Performance of the target tracking algorithm at 10 trials in each direction.

	1	2	3	4	5	6	7	8	9	10	Average ± Standard Deviation
Push	3.0038	3.0040	3.0114	3.0107	3.0143	3.0115	3.0135	3.0114	3.0126	3.0131	3.0106 ± 0.003715
Pull	3.0001	2.9932	3.0000	3.0030	3.0031	2.9999	2.9961	3.0013	2.9998	2.9971	2.9994 ± 0.003093

## Data Availability

The data that support the findings of this study are available on reasonable request from the corresponding author. The data are not publicly available due to privacy or ethical restrictions.
